# Effect of Commercial Trap Design and Location on Captures of *Diocalandra frumenti* (Fabricius) (Coleoptera: Dryophthoridae) on Palm Trees

**DOI:** 10.3390/insects15100738

**Published:** 2024-09-25

**Authors:** Carina Ramos-Cordero, Elena Seris-Barrallo, Sandra Vacas, Vicente Navarro-Llopis, Estrella M. Hernández-Suárez

**Affiliations:** 1 Dirección General de Agricultura, Consejería de Agricultura, Ganadería, Pesca y Soberanía Alimentaria del Gobierno de Canarias, Santa Cruz de Tenerife, Avda. José Manuel Guimerá, 10, Edif. Servicios Múltiples II, Planta 4ª, 38071 Santa Cruz de Tenerife, Spain; ramoscorderocarina@gmail.com (C.R.-C.); seris.elena@inia.csic.es (E.S.-B.); 2 Unidad de Protección Vegetal, Instituto Canario de Investigaciones Agrarias (ICIA), Crta. El Boquerón, s/n, 38270 La Laguna, Spain; 3 Plant Protection Products Unit, National Institute for Agricultural and Food Research and Technology INIA-CSIC, Crta. de La Coruña, km 7.5, 28040 Madrid, Spain; 4 Centro de Ecología Química Agrícola, Instituto Agroforestal del Mediterráneo, Universitat Politècnica de València, Camino de Vera, s/n, 46022 València, Spain; sanvagon@ceqa.upv.es (S.V.); vinallo@ceqa.upv.es (V.N.-L.)

**Keywords:** four-spotted coconut weevil, trapping, monitoring, mass trapping, pest control, kairomones, weevils, trap efficacy, trap design

## Abstract

**Simple Summary:**

The Canary Island palm, *Phoenix canariensis* H. Wildpret, is a resource of great economic and scenic value in the Canary Islands, whose natural palm groves are priority habitats and protected by law. The palms are being severely affected by a small weevil, *Diocalandra frumenti*, whose larvae burrow galleries in the leaves, causing their premature drying and collapse, especially in urban environments. This study focuses on developing an effective trap to capture *D. frumenti*, evaluating several factors such as type, design, colour, height, distance and location of the traps with respect to the palm trees to determine the most efficient configuration. The resulting trap, a green, uncovered Econex^®^ moth trap (Sanidad Agrícola Econex S.L., Murcia, Spain) with ventilation holes and baited with sugar cane and water, proved to be effective in capturing *D. frumenti*. The combination of this trap with a *D. frumenti*-specific pheromone will provide a valuable tool for early detection in areas free of the pest, as well as for monitoring and mass trapping in affected areas. The results of this research will have a significant impact on the protection of the valuable natural palm groves of the islands, benefiting both the economy and the landscape of the Canary Islands.

**Abstract:**

*Diocalandra frumenti* (Fabricius) (Coleoptera: Dryophthoridae) is a weevil present in the Canary Islands, affecting economically important palms such as *Phoenix canariensis* H. Wildpret and its hybrids, for which there were no trapping tools. The larvae cause the main damage by burrowing galleries in the rachis of the leaves, causing premature drying and collapse. To develop an effective trap, six trials were carried out to evaluate the effect of trap type, design, colour, height, distance and location of the trap in relation to the palm tree on *D. frumenti* captures. This study confirms that the Econex^®^ trap, green in colour, without a cover and with two ventilation holes of 2.5 cm in diameter, diametrically opposite each other and at 1 cm from the top of the base of the trap, baited with sugar cane and water, and placed between the first and second ring of green leaves of the palm canopy, is efficient in capturing *D. frumenti*. These results establish a basis for future research focused on the development of a specific trapping system based on semiochemicals to serve as a tool for detection, monitoring and mass trapping of *D. frumenti*.

## 1. Introduction

The four-spotted coconut weevil, *Diocalandra frumenti* [1] [syn. *D. stigmaticollis* Gyllenhal, 1833] (Coleoptera: Dryophthoridae), is native to Southeast Asia [2], from where it has spread to various coastal areas of the Pacific and Indian Oceans [3,4]. Its main hosts include *Cocos nucifera* L. [3,5], *Elaeis guineensis* Jacquin [3] and several economically important ornamental palms such as *Phoenix canariensis* H. Wildpret and its hybrids, *Phoenix dactylifera* L. and *Washingtonia* spp. [3,5,6]. In Asia, there is another species of *Diocalandra* called *D. taitense* (Guérin-Méneville), very similar to *D. frumenti* in description and habit [7], native to the South Pacific and present in Madagascar, the Hawaiian Islands and Brazil [8,9]. The larvae of both species bore into roots, petioles, inflorescences and fruits of palm trees [9]. Adults are 6–8 mm long and 2 mm wide [9,10]. *Diocalandra taitense* is shiny black with four reddish spots on the elytra, while *D. frumenti* is brown with four spots on the elytra, two blackish-brown and two yellowish-brown [4,10] [Supplementary Material, Figure S1]. Life cycles are similar, including an egg stage, which hatches in 8–9 days, a larval stage that lasts 8–10 weeks, a pupal stage that lasts 10–12 days and unfolds without forming a cocoon, and the adult stage [4,9]. Both species cause severe primary damage to roots, leaves and fruit stems, and are one of the causes of premature nut drop in *Areca catechu* L. [7]. Due to the losses caused by both species in Southeast Asia, several studies have been conducted in this region to determine the extent of infestation, seasonal incidence, percentage of losses and management measures in coconut palms and *A. catechu* [11,12].

Only *D. frumenti* is present in Europe and is restricted to the Canary Islands. It first appeared in Maspalomas (San Bartolomé de Tirajana, Gran Canaria) in 1998 on the Canary Island palm (*P. canariensis*) [6,13]. It is currently present on all the islands of the archipelago except El Hierro and La Graciosa [14,15].

The main damage to the palm is caused by the larvae during feeding, digging gal-leries 1–2 mm in diameter in healthy tissue in the basal third of the rachis of green leaves, producing gummy exudations and causing premature drying and collapse of the leaves in the crown of the palm tree, starting from the outer to the inner leaves [6,16] [Supplementary Material, Figure S2].

Indirect damage attributed to *D. frumenti* is that of acting as a vector for the propagation of fungal diseases caused by opportunistic fungi, such as *Nalanthamala* (=*Gliocladium*) *vermoesenii* (Biourge) Schroers, or as a pathway for the entry of plant pathogenic fungi lethal to the palm, such as *Ceratocystis paradoxa* (Dade) C. Moreau, *Fusarium oxysporum* f. sp. canariensis Mercier & Louvet or *Thielaviopsis radicicola* (Bliss) Z.W. De Beer & W.C. Allen (previously identified as *Thielaviopsis punctulata*) [13,17,18].

The Canary Island palm tree is of enormous value in the Canary Island archipelago. It represents a key element of the economy of some productive sectors, such as those dedicated to the production of handicrafts and the production of guarapo (drink from palm sap) and palm syrup. Furthermore, it has botanical, scientific, ecological and scenic value, as the natural palm groves constitute a habitat of Community interest 9370 Palmerales de Phoenix, included in the Habitats Directive (D92/43/CEE), and designated as a priority habitat [19]. These are the main reasons that have led the Canary Island palm tree to be considered a plant symbol of the Autonomous Region of the Canary Islands, according to Law 7/1991, of 30 April, on symbols of nature for the Canary Islands [20], and as such, it is protected by law [21].

In 2007, due to the presence of *D. frumenti* on the islands of Gran Canaria, Lanzarote, Fuerteventura and Tenerife, the Department of Agriculture, Livestock, Fisheries and Food of the Canary Island Government issued an Order of 29 October 2007 [22]. This order included the proper pruning of palm trees and the management of their waste, along with the use of chlorpyrifos 48% EC and imidacloprid 20% SL as chemical control methods. However, since 2018, the use of imidacloprid on palm trees has been unauthorised according to Commission Implementing Regulation (EU) 2018/783 [23], and the same occurred with chlorpyrifos in 2020, according to Commission Implementing Regulation (EU) 2020/18 [24]. The aim was to prevent the pest from spreading to the natural palm groves of the islands, given that *D. frumenti* is mainly found in urban environments. With the entry into force of Royal Decree 1311/2012 of 14 September, it is mandatory to promote integrated pest management (IPM) strategies that minimise costs, side effects and risks to the environment [25,26] and that include pest monitoring and the establishment of action thresholds, where pesticides play an important role, especially in cryptic species [26].

The importance of having an effective trapping system for *D. frumenti* is crucial, especially considering that, after reviewing the existing literature, there was no known trapping system developed for this pest. An effective trapping system consists of the trap, attractants and a retentive element [27,28]. Trap effectiveness depends on several factors such as trap type [29,30,31,32,33,34], trap design [35,36,37], trap colour [34,38,39,40,41,42,43,44,45], trap shape [42,44,46,47], placement height [39,48,49,50,51], trap location [43,50,52] and application density [31,53,54].

For *D. frumenti*, unlike other coleopteran palm borer pests such as *Rhynchophorus ferrugineus* Olivier (Faleiro, 2006), *Oryctes* spp. (Coleoptera: Scarabaeidae) (Bedford et al., 2015; Faleiro, 2006; Rochat et al., 2004) or *Jebusaea hammerschmidti* (Reiche) (Coleoptera: Cerambycidae) [55,56,57], no monitoring tools have been developed. The implementation of a trap that enables early detection, monitoring, and mass capture is essential for managing this pest, which affects palm trees in both urban areas and natural environments. This study began with the evaluation of different commercial traps used for other pests. Regarding attractants, tests were conducted using sugar cane as bait, as *Diocalandra* sp. is a significant pest of sugar cane in countries like China, where it appeared in the 1980s, causing severe damage to the plant roots, leading to root rot, wilting and lodging of the plants [58].

It is important to have a management and control tool for the early detection, monitoring and mass trapping of *D. frumenti*. Therefore, the purpose of this research is to test the effect of trap type, design, colour, height, placement distance and location of the trap with respect to the palm tree on *D. frumenti* captures.

## 2. Materials and Methods

In this study, a total of six trials were carried out, based on the results of two initial tests, in which various aspects of trap design and location were evaluated for the capture of *D. frumenti*. Table 1 lists the initial tests, and Table 2 presents the definitive trials on the configuration and position of the trapping system. Both tables include the objective, the location, the date, the treatments evaluated and the scheme of each trial.

### 2.1. Initial Tests

The research, initiated in 2013, revealed that there was no published information on the trapping of *D. frumenti*. Due to this lack of information, a series of initial tests were carried out to determine the combination of trap, attractants and retentive substance that would capture *D. frumenti*.

#### 2.1.1. Study Areas

Two initial tests were conducted in the Canary Islands, one in Tenerife and the other in Gran Canaria, at two specific locations: the landscaped areas of the Eureka Apartments, located in TenBel in Arona (Tenerife) (28°00′25″ N, 16°38′39″ W, 23 m a.s.l.), and the Parque Romano in Las Palmas de Gran Canaria (Gran Canaria) (28°07′29″ N, 15°25′38″ W, 5 m a.s.l.). The tests focused on the Canary Island palm and its hybrids, selecting specimens with stipules between 3 and 5 m in height to ensure comparison between the study subjects. The test areas were selected based on the number of palms present, their uniformity in height and other agronomic factors such as the type of irrigation and solar radiation received by the palms. Before the start of each trial, the area was trapped to evaluate the level of infestation of the palms by *D. frumenti*.

#### 2.1.2. Description of the Tests

In the first test, two commercial traps were evaluated to determine their effectiveness in controlling *D. frumenti*. The traps evaluated were (Table 1): (a) a 15-L bucket trap, typically used for trapping *R. ferrugineus* (Ao Midori Biocontrol S.L., Barcelona, Spain), which had four 6 cm diameter holes positioned in diametrically opposed pairs near the top of the bucket base, and three holes of the same diameter in the lid (hereafter, bucket trap); and (b) a green Econex^®^ trap for lepidoptera, modified for this trial. This trap was used without the top lid and was modified with two ventilation holes of 2.5 cm in diameter, placed diametrically opposite each other 1 cm from the top of the trap base (hereafter, green Econex^®^). These modifications were made on the basis of previous tests carried out by our team, the results of which have not been published (Seris-Barrallo, pers. comm.). The bucket trap was baited with 1 kg of sugar cane and water, while the green Econex^®^ trap was baited with 200 g of sugar cane and water, following the optimal trapping protocols for other pests such as *R. ferrugineus* [54,59,60]. The bucket trap was hung on the first green leaf ring of the crownshaft and the green Econex^®^ trap was inserted between the first and second green leaf ring of the crownshaft.

In a second test, the need to add water to the trap and its possible retentive effect on the capture of adult *D. frumenti* was evaluated. Captures recorded in the green Econex^®^ trap were employed as the best design obtained in the previous test. The traps were baited with two 15 cm long sugar cane fragments, cut lengthwise, in the following combinations (Table 1): (a) sugar cane only; (b) sugar cane and 500 mL water; and (c) the base of the trap was coated inside with Soveurode^®^ (Witasek), an adhesive spray used for insect control. Once the glue dried, the trap was baited with sugar cane and 500 mL of water. The traps were placed between the first and second leaf rings of the crownshaft.

### 2.2. Trials to Improve the Effectiveness of the Trapping System

#### 2.2.1. Study Areas

The trial areas were located in the Canary Islands, specifically on the island of Gran Canaria, covering five different locations: the Don Benito urban park in Las Palmas de Gran Canaria (28°06′45″ N 15°25′47″ W, at 101 m a.s.l.); three locations in San Bartolomé de Tirajana: an urban palm grove in Campo Internacional (27°45′38″ N 15°35′08″ W, at 30 m a.s.l.); the alignment of palm trees in the Avenida Tour Operador Tui (27°45′39″ N 15°35′16″ W, at 30 m a.s.l.); and in the botanical garden Parque Tony Gallardo (27°44′47″ N 15°35′55″ W, at 7 m a.s.l.); and the landscaped areas of the company Vidrieras Canarias S.A. in Telde (27°58′47″ N 15°23′12″ W, at 39 m a.s.l.). The trials followed the same methodology described in Section 2.1.1 regarding the selection of palm specimens, their uniformity, and the assessment of *D. frumenti* infestation levels.

#### 2.2.2. Trap Design

Trap type trial 1 was carried out to evaluate the trapping efficacy of three commercially available traps (Table 2): (a) the abovementioned green Econex^®^ trap; selected for its efficacy in the initial tests; (b) Crosstrap^®^ trap for Coleoptera interception during flight (Sanidad Agrícola Econex S.L., Murcia, Spain); and (c) Theysohn^®^ slot trap for bark beetles (Theysohn Group, Salzgitter, Germany), used for the capture of forest pests, mainly weevils of the family Scolytidae. The green Econex^®^ trap was placed between the first and second ring of green leaves of the crownshaft, the Crosstrap^®^ trap between the second and third ring of green leaves of the crownshaft and the Theysohn^®^ trap, due to its large size, occupied the entire base of the crownshaft.

Trial 2, trap design, aimed to compare *D. frumenti* captures recorded with three green Econex^®^ trap designs (Table 2): (a) without top cover and modified with two ventilation holes 2.5 cm in diameter diametrically opposite each other and 1 cm from the top of the trap base; (b) without cover and no hole modification; and (c) with cover and modified with two ventilation holes 2.5 cm in diameter and diametrically opposite one another and 1 cm from the top of the trap base.

Trial 3, trap base colour, aimed to compare *D. frumenti* captures recorded using three colours of Econex^®^ trap base, without top cover and modified with two ventilation holes (Table 2): (a) green, (b) transparent and (c) white. The selection of the colours white and transparent was based on the hypothesis that these colours might have different levels of attraction for *D. frumenti* compared to green.

#### 2.2.3. Trap Location

Trial 4, trap placement height on the palm tree, was carried out with the aim of comparing the captures recorded in traps placed at three different heights on the palm tree (Table 2): (a) on the stipe, 40 cm above the ground, (b) on the stipe, at mid-height, and (c) on the crownshaft, inserted between the first and second ring of green leaves.

Trial 5, trap placement distance from the palm tree, was replicated in two different locations: the first at Parque Tony Gallardo and the second in Vidrieras Canarias S.A. The objective in both replicates was to test the effect on *D. frumenti* captures of placing the trap at different distances from the palm tree (Table 2): (a) 0 m, (b) 3 m and (c) 15 m, placed on a pole 1.20 m from the ground.

Trial 6, location of the trap with respect to the palm tree, was aimed at testing the effect on *D. frumenti* captures of placing the trap in different locations regarding the palm tree (Table 2): (a) at 0 m, at the crownshaft, (b) at 5 m, on a pole at the height of the crownshaft and (c) at 5 m, 40 cm above the ground.

### 2.3. Trial Procedure

The initial tests and trials were organised according to a randomised complete block design with the number of repetitions per trial listed in Table 2. To minimise the effect of position, weekly intrablock rotation of the traps was performed. The traps were placed between the first and second rings of green leaves in the crownshaft, oriented to the south, and baited with two 15 cm long sugar cane fragments, cut lengthwise, and 500 mL of water, based on the results obtained in the initial tests.

The traps were checked weekly to renew their contents and extract the captured individuals, which were then counted and sexed in the laboratory. Sexing was carried out using a Nikon^®^ SMZ645 stereo microscope (Melville, NY, USA) to determine whether the treatments evaluated influenced the sex ratio of *D. frumenti*. Sex identification was determined by observation of the adult rostrum, a diagnostic character that distinguishes females (thinner, shinier and apically more arched rostrum) from males (wider, rougher textured and apically uncurved rostrum) [61]. In all trials in this study, all adults captured in each trap were counted and sexed.

### 2.4. Statistical Analysis

The data analysis for the first test was conducted using a Student’s *t*-test to compare the means of the experimental groups. Before applying the test, the normality of the data and the homogeneity of variances were verified. The *t*-test was used to determine if there were significant differences between the treatments evaluated, with a significance level set at *p* < 0.05. For the second test and the six trials, a multifactorial analysis of variance (ANOVA) was performed to identify statistically significant differences in the *D. frumenti* captures recorded in each trial. Prior to each analysis, normality and the homogeneity of variances were checked, and when necessary, data were normalised using a log(x + 1) transformation. The Tukey’s–HSD multiple range test (*p* < 0.05) was applied to differentiate the mean differences among the parameters across all treatments.

All statistical analyses were performed using Statgraphics^®^ Centurion XIX for Windows, and the figures were created with Microsoft Office Excel 2019. Data are presented as untransformed means ± standard error of the mean.

## 3. Results

### 3.1. Overall Results

In all tests and trials, the sex ratio shows a slight predominance of females over males. The values obtained in the Student’s *t*-test show that there were no statistically significant differences between the means of males and females in any of the tests or trials (*p*-values > 0.05), suggesting that, in general terms, the capture of males and females was balanced (Table 3).

### 3.2. Initial Tests

**Figure 1:**
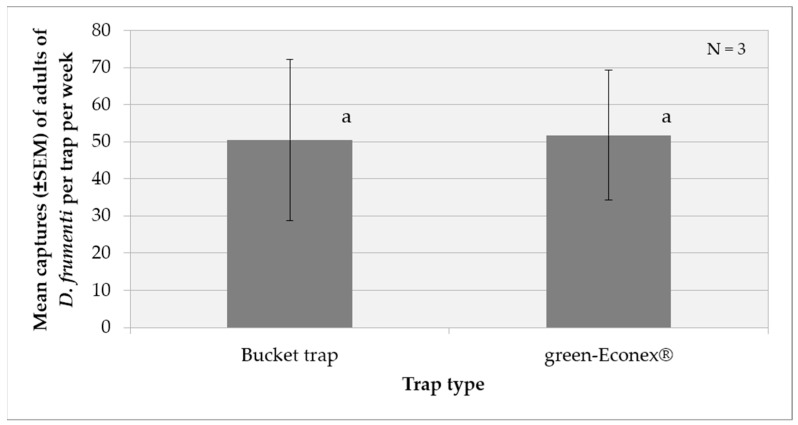
Mean captures (±SEM) of adult *D. frumenti* per trap per week in two different trap types baited with sugar cane and water in Apartamentos Eureka (TenBel, Tenerife) from 5 June to 25 July 2014. N = number of replicates. The comparison of means was performed using a two-factor ANOVA. Post hoc analyses using Tukey’s test could not be performed because, for the factor “week”, one group had fewer than two cases, and for the factor “treatment”, there were fewer than three groups.

In the first test, the two-factor analysis of variance (ANOVA) performed for the transformed catch variable (log(x + 1)) showed no significant effect of the week factor (F(4, 19) = 1.220, *p* = 0.335) nor of the treatment factor (F(1, 19) = 0.009, *p* = 0.923). The interaction between week and treatment was also not significant (F(3, 19) = 1.328, *p* = 0.295). These results indicate that there are no statistically significant differences in *D. frumenti* captures between the bucket trap and green Econex^®^ traps across weeks (Figure 1).

**Figure 2:**
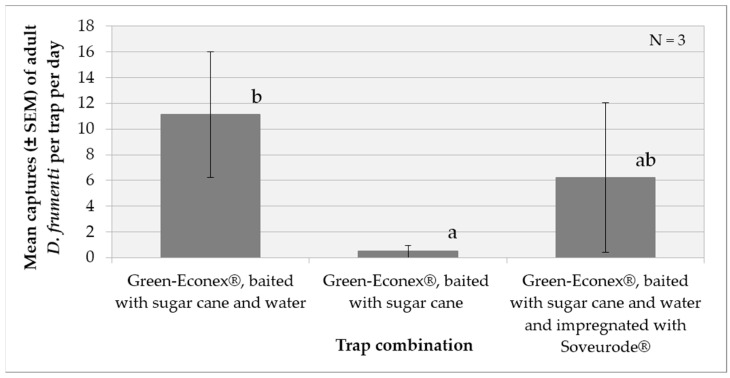
Mean captures (±SEM) of adult *D. frumenti* per trap per day in two different trap types baited with sugar cane and water in Apartamentos Eureka (TenBel, Tenerife) from 05 June to 25 July 2014. N = number of replicates. The comparison of means was performed using the Student’s *t*-test for independent samples. Means with equal letters do not differ significantly (*p* < 0.05).

In the second test, the analysis of variance (ANOVA) for the variable captures per trap per day, transformed as (log(x + 1)), revealed significant differences between the treatment factors (F = 4.31, *p* = 0.0488) and repetition (F = 5.41, *p* = 0.0287). The week factor does not show a significant effect, with F(1, 12) = 0.9 and *p* = 0.3605. However, no significant interaction was detected between these factors (F = 0.78, *p* = 0.5637). The multiple range analysis using Tukey’s HSD method (*p* = 0.05) showed that the treatment “green Econex^®^ trap, baited with sugar cane and water” is significantly different from the treatment “green Econex^®^ trap, baited with sugar cane” treatment. The treatment “green Econex^®^ trap, baited with sugar cane and water, and internally impregnated with Soveurode^®^” does not significantly differ from the other two treatments (Figure 2).

### 3.3. Trials to Improve Trapping System Efficiency

#### 3.3.1. Trap Design

##### Effect of Trap Type

**Figure 3:**
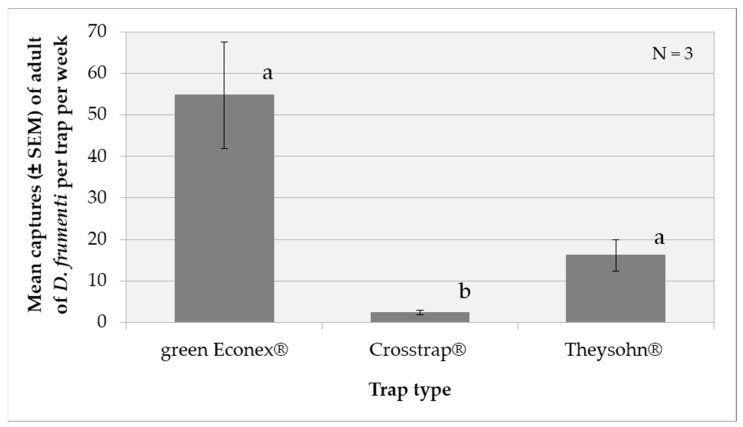
Mean captures (±SEM) of adult *D. frumenti* per trap per week in three different trap types baited with sugar cane and water in Parque Don Benito (Las Palmas de Gran Canaria, Gran Canaria) from 18 June to 30 July 2015. N = number of replicates. Data were analysed using multifactorial ANOVA. Comparison of means was performed using Tukey’s HSD multiple range test (*p* = 0.05). Means with equal letters do not differ significantly (*p* < 0.05).

The analysis of variance (ANOVA) for the transformed captures variable, log(x + 1), shows that both the treatment factor (F(2, 20) = 7.36, *p* = 0.004) and the repetition factor (F(2, 20) = 10.86, *p* = 0.0006) have significant effects. The week factor does not show a significant effect, with F(5, 20) = 0.82 y *p* = 0.5522. However, no significant interactions were found between the factors analysed. The multiple range tests analysis for log(x + 1) by treatment using Tukey’s HSD method (*p* = 0.05) reveals that the green Econex^®^ treatment has a significantly higher capture performance compared to Crosstrap^®^ (*p* < 0.05), while there are no significant differences between green Econex^®^ and Theysohn^®^. Crosstrap^®^, in turn, is significantly less effective than Theysohn^®^ (Figure 3).

##### Effect of Trap Design

**Figure 4:**
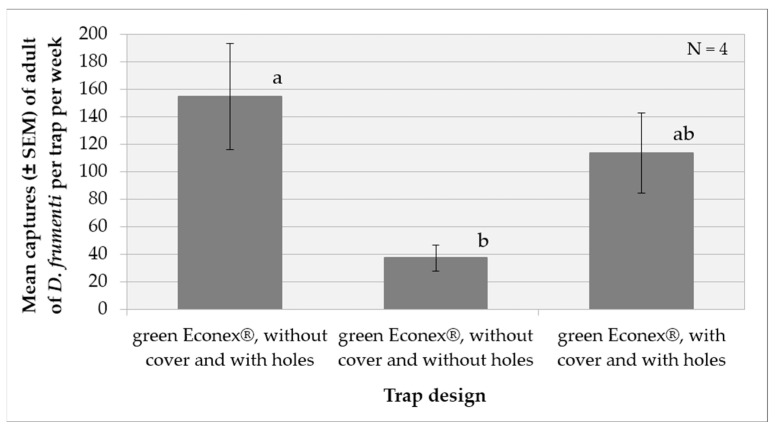
Mean captures (±SEM) of *D. frumenti* adults per trap and week in three different trap designs, baited with sugar cane and water in Campo Internacional (San Bartolomé de Tirajana, Gran Canaria), from 3 November to 1 December 2014. N = number of replicates. Data were analysed by multifactorial ANOVA. Comparison of means was performed using Tukey’s HSD multiple range test (*p* = 0.05). Means with equal letters do not differ significantly (*p* < 0.05).

The analysis of variance (ANOVA) for the transformed captures variable, log(x + 1), shows that the factor weeks (F(3, 17) = 3.26, *p* = 0.0471) and the factor treatment (F(2, 17) = 5.42, *p* = 0.0151) have significant effects, while the factor repetition did not show a significant impact (F(3, 17) = 1.69, *p* = 0.2067). No significant interactions were detected between the evaluated factors. The multiple range analysis for log(x + 1) by treatment using Tukey’s HSD method (*p* = 0.05) revealed that the treatment “green Econex^®^, without cover and with holes” is significantly more effective than “green Econex^®^, without cover and without holes” (*p* < 0.05), while no significant differences were observed between “green Econex^®^, without cover and with holes” and “green Econex^®^, with cover and with holes”. “green Econex^®^, without cover and without holes” is significantly less effective than “green Econex^®^, with cover and with holes” (Figure 4).

##### Effect of Trap Base Colour

**Figure 5:**
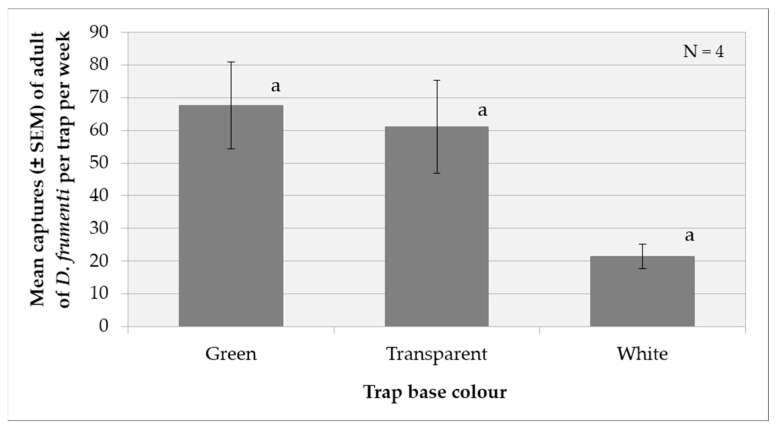
Mean captures (±SEM) of *D. frumenti* adults per trap and week in traps with three different base colours, baited with sugar cane and water in Campo Internacional (San Bartolomé de Tirajana, Gran Canaria), from 03 November to 01 December 2014. N = number of replicates. Data were analysed by multifactorial ANOVA. Comparison of means was performed using Tukey’s HSD multiple range test (*p* = 0.05). Means with equal letters do not differ significantly (*p* < 0.05).

The analysis of variance (ANOVA) for the transformed capture variable, log(x + 1), indicates that the week factor (F(3, 16) = 10.02, *p* = 0.0006) has a significant effect on captures, while the factors repetition (F(3, 16) = 3.01, *p* = 0.0611) and treatment (F(2, 16) = 2.93, *p* = 0.0824) do not show significant differences. Among the interactions, the week × treatment interaction is significant (F(6, 16) = 2.88, *p* = 0.0423), while the others are not. The multiple range analysis using the Tukey HSD method at 95% reveals that there are no significant differences between the treatments, as they all group into the same homogeneous group. This suggests that, although the “green” treatment has a higher mean, the differences do not reach statistical significance (Figure 5). Additionally, separate analyses by week, using the Tukey HSD method, confirmed that there are no significant differences between treatments in any of the weeks evaluated.

#### 3.3.2. Trap Location

##### Effect of Trap Placement Height on the Palm Tree

**Figure 6:**
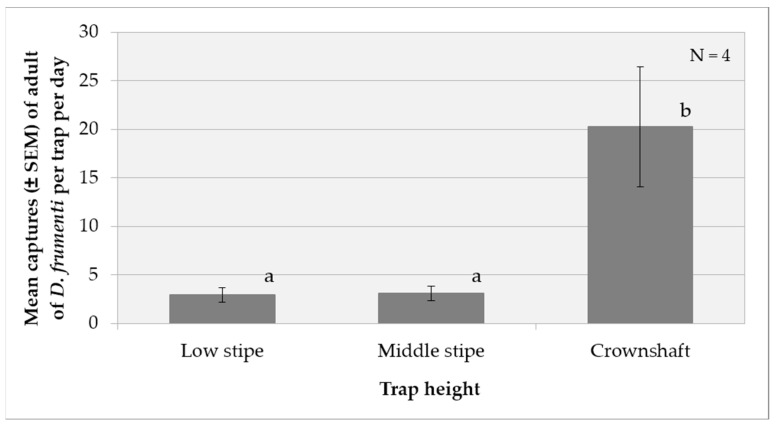
Mean captures (±SEM) of *D. frumenti* adults per trap and day in traps placed at three different heights in the palm tree, baited with sugar cane and water at Avenida Tour Operador Tui (San Bartolomé de Tirajana, Gran Canaria), from 27 October to 18 November 2016. N = number of replicates. Data were analysed using multifactorial ANOVA. Means were compared using Tukey’s HSD multiple range test (*p* = 0.05). Means with equal letters do not differ significantly (*p* < 0.05).

The analysis of variance (ANOVA) for the transformed variable captures per day, log(x + 1), shows that the treatment factor has a significant effect (F(2, 12) = 5.70, *p* = 0.0182) on daily captures, while the week factor (F(2, 12) = 3.51, *p* = 0.063) and the repetition factor (F(3, 12) = 0.25, *p* = 0.859) do not show significant differences. No significant interactions between the factors were detected. Multiple range tests for log(x + 1) by treatment using Tukey’s HSD method (*p* = 0.05) reveal that the “crownshaft” treatment is significantly more effective compared to “low stipe” and “middle stipe” (*p* < 0.05) (Figure 6).

##### Effect of Trap Placement Distance on the Palm Tree

**Figure 7:**
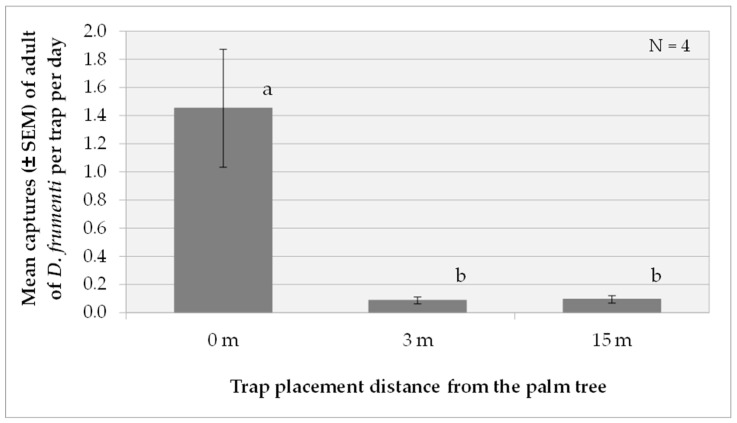
Mean captures (±SEM) of *D. frumenti* adults per trap and day in traps placed at three different distances from the palm tree, baited with sugar cane and water in the Parque Tony Gallardo (San Bartolomé de Tirajana, Gran Canaria), from 27 October to 18 November 2016. N = number of replicates. Data were analysed using multifactorial ANOVA. Means were compared using Tukey’s HSD multiple range test (*p* = 0.05). Means with equal letters do not differ significantly (*p* < 0.05).

The analysis of variance (ANOVA) for the variable captures per day transformed, log(x + 1), indicates that the treatment factor has a highly significant effect on daily captures (F(2, 12) = 55.68, *p* < 0.0001). On the other hand, the factors week (F(2, 12) = 0.09, *p* = 0.9142) and repetition (F(3, 12) = 2.88, *p* = 0.0801) do not show significant effects. Regarding interactions, only the interaction between repetition and treatment is significant (F(6, 12) = 4.43, *p* = 0.0137). Multiple range tests for log(x + 1) by treatment using Tukey’s HSD method (*p* = 0.05) reveal that the treatment at 0 m is significantly more effective than the treatments at 3 m and 15 m (*p* < 0.05) (Figure 7).

Additionally, non-parametric analyses were performed using the Kruskal–Wallis test for each replicate separately. The results showed that only in replicates 1 and 4 were there statistically significant differences in daily catches between treatments at different distances (0 m, 3 m and 15 m) (replicate 1: ꭕ^2^ = 6.720, gl = 2, *p* = 0.035; replicate 4: ꭕ^2^ = 6.720, gl = 2, *p* = 0.035). In replicates 2 and 3, no significant differences were found (replicate 2: ꭕ^2^ = 5.793, gl = 2, *p* = 0.055; replicate 3: ꭕ^2^ = 5.915, gl = 2, *p* = 0.052). These results suggest that the relative performance of treatments varies with replication, which justifies the need to consider the interaction between replication and treatment in the analysis.

**Figure 8:**
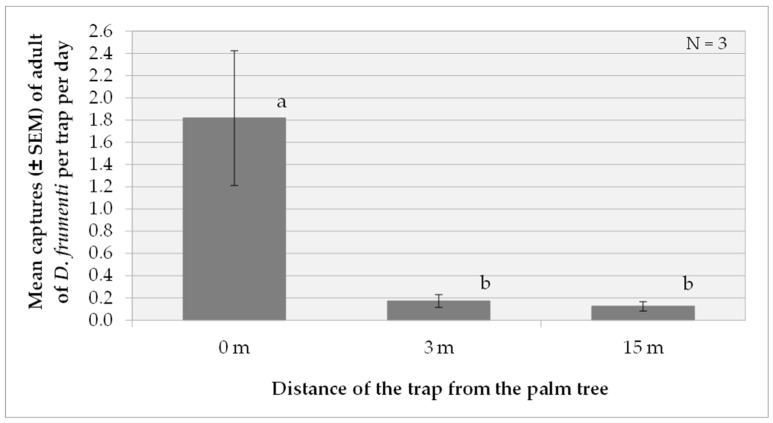
Mean captures (±SEM) of *D. frumenti* adults per trap and day in traps placed at three different distances from the palm tree, baited with sugar cane and water in Vidrieras Canarias S.A. (Telde, Gran Canaria), from 11 to 31 October 2016. N = number of replicates. Data were analysed by multifactorial ANOVA. Comparison of means was performed using Tukey’s HSD multiple range test (*p* = 0.05). Means with equal letters do not differ significantly (*p* < 0.05).

Comparing the results of this analysis with those obtained at the previous location, a consistent pattern is observed in the effectiveness of the treatment at 0 m, which again stands out as the most effective in terms of daily captures (F(2, 18) = 194.32, *p* < 0.0001). In both locations, this treatment showed significant differences compared to the treatments at 3 m and 15 m. However, it is important to note that in this new location, the factor “week” also showed a significant effect on captures (F(2, 18) = 4.3, *p* = 0.0297), which could indicate a more pronounced temporal influence in this new location (Figure 8).

##### Effect of Trap Location Relative to the Palm Tree

A total of 173 adults of *D. frumenti* were captured in this trial, all of them in the traps placed in the palm flange (19.22 ± 6.41 adults/trap/week). No individuals were captured in the traps placed at a distance of 5 m from the palm tree, on a pole at the height of the crownshaft or 40 cm above the ground. This confirms the need to place the traps in the palm tree to maximise captures.

## 4. Discussion

The present study focused on evaluating the effect of commercial trap type, design, colour, height, distance and location of the trap with respect to the palm tree on *D. frumenti* captures, with the aim of generating an efficient trap prototype.

In the trials carried out, no statistically significant differences were detected in the number of females and males of *D. frumenti* captured. However, a slight bias towards females was observed in the mean sex ratio, which is of particular importance for a reduction of the reproductive capacity of the population. This same pattern has been documented in *R. ferrugineus*, as reported in previous studies [62,63,64]. However, it is important to note that this study did not determine the reproductive status of the females captured, i.e., whether they were virgins, mated or had laid eggs. This aspect will be considered in future studies to more accurately assess population dynamics and the effectiveness of control strategies.

The analysis of the trap design showed that, in the evaluation of different trap types, the experimental green Econex^®^ trap stood out as the most effective in catching *D. frumenti* compared to the Crosstrap^®^ and Theysohn^®^ traps. According to Allison and Redak [65], the Crosstrap^®^ and Theysohn^®^ traps are specifically designed to capture forest beetles (Scolitidae) [66,67]. However, our observations indicate that *D. frumenti* make short flights and move along the palm tree by walking, suggesting that these types of traps may not be as effective in capturing them. Furthermore, while the Crosstrap^®^ trap was anchored to the canopy between the second and third ring of green leaves, and the Theysohn^®^ trap occupied the entire base of the canopy due to its large size, the green Econex^®^ trap was strategically placed between the first and second ring of green leaves of the canopy, where the adults of *D. frumenti* are usually found in the palm, according to Salomone et al. [6]. In other words, in addition to being more effective in capturing weevils, the green Econex^®^ trap allows, from an operational point of view, relatively easy placement in the crownshaft due to its size and design.

Likewise, traps with wet collection containers retain more insects than those with dry ones, reducing the probability of escape of the captured insects. Regarding Crosstrap^®^, several studies have shown that treating the slats with non-stick material such as Teflon^®^ or Fluon^®^ increases their effectiveness in capturing cerambycids [30,35,51,68,69,70,71]. However, in our study, the slats were not treated, which could have reduced the number of *D. frumenti* captures. In addition, the small size of the slots in the Theysohn^®^ trap limits the emission of the attractants [33,72,73], which may have reduced their efficacy in capturing *D. frumenti*. Although little is known about how odour plume structure varies between different types of interception traps, it is likely that these differences in plume structure contribute to the observed variations in trap performance [73,74].

Although our initial study showed that the green Econex^®^ trap was significantly more effective in capturing *D. frumenti*, it is possible that other factors, such as the specific location of the traps and the flight behaviour of the insects, also influence the effectiveness of the traps. Therefore, we propose conducting additional studies that include the analysis of the flight and movement behaviour of *D. frumenti*, and tests under different environmental conditions to evaluate how factors such as temperature, humidity and sun exposure affect the effectiveness of the traps.

In our research, it was observed that the green Econex^®^ trap, without a top cover and with two ventilation holes, was the design that captured the highest number of *D. frumenti* adults. This design is the one with the largest ventilation surface, which allows an adequate release of attractants into the air. *Diocalandra frumenti* did not show a clear preference for the colour of the trap, as is the case with other insects such as *Rhynchophorus palmarum* L. (Coleoptera: Curculionidae) [75], *Metamasius hemipterus* sericeus (Olivier) (Coleoptera: Dryophthoridae) [76] or *Xyleborus glabratus* Eichhoff (Coleoptera: Curculionidae: Scolytinae) [31,49].

The study of the location of the trap in relation to the palm reveals that the highest captures of *D. frumenti* are obtained when the trap is placed between the first and second ring of green leaves of crownshaft. Although, logistically, it may be difficult to place the trap at this height, requiring the use of ladders or vehicles with lifting platforms, we observed that captures at the crownshaft were seven times more numerous than those recorded at ground level. Proper placement of the trap could make the difference between detecting or not detecting a *D. frumenti* population in areas considered to be uninfested. Regarding trap placement distance, the highest captures were recorded when the trap was placed on the stipe of the palm, with almost zero captures detected as the trap placement distance increased.

This finding agrees with that obtained by Aldryhim and Al-Bukiri for *R. ferrugineus* [77], where traps set in shaded areas near infested palm trees and with moist soil recorded the highest captures. The position of the trap is one of the factors affecting trap catches, and this fact has been demonstrated in other weevils such as *Cosmopolites sordidus* Germar (Coleoptera: Curculionidae) [78]. In the case of *D. frumenti*, captures were only recorded when the trap was placed between the first and second ring of green leaves of the crownshaft, and no captures were recorded when the trap was placed 5 m away from the palm, on a pole at the height of the crownshaft or at ground level.

Despite its usefulness, this kairomone-based trapping system has several limitati-ons. On the one hand, installing the traps on the crownshaft of palm trees requires the use of ladders or vehicles with lifting platforms, and this work at height increases the risk for the operators. On the other hand, like other food baits, sugar cane has a low attraction power by itself and decomposes quickly, reducing the effectiveness of the traps [79,80]. Parallel to this research, Vacas et al., carried out the identification and synthesis of the *D. frumenti* pheromone, providing a solid foundation for future trials and the development of more effective control strategies [81].

In this study, we tested several commercial traps, and the green Econex^®^ trap, with a number of modifications, showed the best performance in catching *D. frumenti*. However, it is still an adapted trap and not one specifically designed for *D. frumenti* control.

Based on our results, future studies will focus on designing a specific trap for *D. frumenti* made from biodegradable material, with a low visual impact design that blends perfectly with the palm tree. This trap will not use insecticide as a retentive for *D. frumenti* adults, so it will not be considered a phytosanitary product. The installation and removal of the trap on the palm tree will be possible using a telescopic pole, significantly reducing the time spent on this action and the risk to the operator by avoiding work at height. The trap will incorporate the *D. frumenti* pheromone as an attractant and use absorbent gels to prolong water retention and increase the service period of the traps in the field.

However, the simple use of traps may not be sufficient to effectively limit the pest population. Therefore, future actions should focus on an integrated pest management (IPM) approach, which includes other measures such as biological control, the use of specific insecticides and appropriate cultural practices. This comprehensive approach would improve early detection, monitoring, and mass capture of this pest, both in pest-free areas and infested areas.

## 5. Conclusions

Our preliminary study indicates that trap design, height, distance and position of the trap in relation to the palm are key factors influencing the capture of *D. frumenti*. In particular, the experimental green Econex^®^ trap, without a cover and with two diametrically opposed ventilation holes of 2.5 cm in diameter and each located 1 cm from the upper edge of the base of the trap, baited with sugar cane and water, and placed between the first and second ring of green leaves of the palm tree’s canopy, has proven to be the most efficient in capturing *D. frumenti*.

Building on these findings, future studies will focus on designing a specific trap for *D. frumenti* made from biodegradable material with a low visual impact design that blends with the palm tree. This trap will not use insecticide, so it will not be considered a phytosanitary product. The installation and removal of the trap will be possible using a telescopic pole, reducing time and risk for the operator. Additionally, the trap will incorporate the *D. frumenti* pheromone as an attractant and use absorbent gels to prolong water retention and increase the service period in the field.

## Figures and Tables

**Table 1:** Summary of the initial tests carried out, their location, test period and treatments evaluated.

Initial Test	Test Area and Test Period	Treatments Tested	
1. Evaluation of different traps in the capture of *D. frumenti*	Apartamentos Eureka (Arona, Tenerife) 28°00′25″ N 16°38′39″ W 23 m a.s.l. Landscaped area of 1.29 ha. Trial period: 5 weeks (5 June–25 July 2014) N° of simultaneous repetitions: 3.	Traps: (a) Bucket type, 15-L capacity, black in color, with ventilation holes in the base and lid, baited with 1 kg of sugar cane and water, and hung from the first green leaf ring of the palm’s crownshaft; (b) green Econex^®^, without a cover and with ventilation holes, baited with 200 g of sugar cane and water, and placed inserted between the first and second green leaf rings of the palm’s crownshaft.	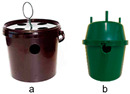
2. Evaluation of the need to add water to the trap in the capture of *D. frumenti*	Parque Romano (Las Palmas de Gran Canaria, Gran Canaria) 28°07′29″ N 15°25′38″ W 5 m a.s.l. Urban park of 1.45 ha Trial period: 2 weeks (29 July–14 August 2013) N° of simultaneous repetitions: 3.	Green Econex^®^ trap baited with (a) sugar cane and water; (b) sugar cane; (c) sugar cane, water and impregnated internally with Soveurode^®^.	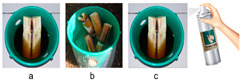

**Table 2:** Summary of the trials carried out, their location, trial period and treatments evaluated.

	**Trial**	**Trial Area, Trial Period and n° of Repetitions**	**Treatments Tested**	
Trap design	1. Effect of trap type on *D. frumenti* captures	Parque Don Benito (Gran Canaria) 28°06′45″ N 15°25′47″ W 101 m a.s.l. Urban park of 5045 m^2^ Test period: 6 weeks (18 June–30 July 2015) N° of simultaneous repetitions: 3.	Traps: (a) green Econex^®^; (b) Crosstrap^®^; (c) Theysohn^®^.	** 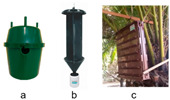 **
	2. Effect of trap design on *D. frumenti* captures	Campo Internacional (Gran Canaria) 27°45′39″ N 15°25′16″ W 30 m a.s.l. Urban palm grove of 8503 m^2^. Trial period: 4 weeks (3 November–1 December 2014) N° of simultaneous repetitions: 4	Econex^®^ trap (a) without top cover and with ventilation holes; (b) without cover and ventilation holes; (c) with cover and ventilation holes.	** 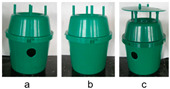 **
	3. Effect of trap base colour on *D. frumenti* captures	Campo Internacional (Gran Canaria) 27°45′39″ N 15°25′16″ W 30 m a.s.l. Urban palm grove of 8503 m^2^. Trial period: 4 weeks (3 November–1 December 2014) N° of simultaneous repetitions: 4	Econex^®^ trap, with base colour (a) green; (b) transparent; (c) white.	** 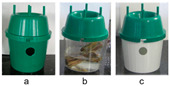 **
Location of the trap	4. Effect of trap height on *D. frumenti* captures	Avenida Tour Operador Tui (Gran Canaria) 27°45′39″ N 15°35′16″ W 30 m a.s.l. 583 m palm alignment Test period: 3 weeks (27 October–18 November 2016) N° of simultaneous repetitions: 4	Green Econex^®^ trap, placed (a) in the stipe, 40 cm above the ground; (b) in the stipe, at mid-height; (c) in the crownshaft, between the first and second ring of green leaves;	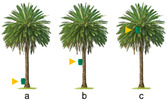
	5. Effect of trap placement distance from the palm tree on *D. frumenti* captures.	Parque Tony Gallardo (Gran Canaria) 27°44′47″ N 15°35′55″ W 7 m a.s.l. Botanical Garden of 8.93 ha Trial period: 3 weeks (27 October–18 November 2016) N° of simultaneous repetitions: 4	Green Econex^®^ trap, placed on a pole 1.20 m above the ground at (a) 0 m; (b) 3 m; (c) 15 m, with respect to the palm tree.	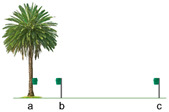
		Vidrieras Canarias S.A. (Gran Canaria) 27°58′47″ N 15°23′12″ W 39 m a.s.l. Plot of 6.46 ha Trial period: 3 weeks (11–31 October 2016) N° of simultaneous repetitions: 4		
	6. Effect of trap location on catches of *D. frumenti*	Vidrieras Canarias S.A. (Gran Canaria) 27°58′47″ N 15°23′12″ W 39 m a.s.l. Plot of 6.46 ha Trial period: 3 weeks (7–28 March 2017) N° of simultaneous repetitions: 3	Green Econex^®^ trap, placed (a) at 0 m, on the crownshaft; (b) at 5 m, on a post at crownshaft height; (c) at 5 m, on a post 40 cm above the ground.	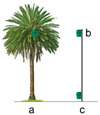

**Table 3:** Summary of sex ratio and statistical analysis across different tests and experiments.

Test/Trial	Male		Female		Sex Ratio	Levene’s Test		Student’s *t*-Test		
	N°	%	N°	%	♂:♀	F	*p*	t	d.f.	*p*
Test										
1	676	47.11	759	52.89	1:1.12	0.101	0.752	−0.320	48	0.750
2	488	50.31	482	49.69	1:0.99	0.010	0.923	0.014	34	0.989
Trials										
1	609	46.28	707	53.72	1:1.16	0.168	0.683	−0.304	106	0.762
2	2318	48.56	2455	51.44	1:1.06	0.138	0.711	−0.171	92	0.865
3	2249	48.65	2374	51.35	1:1.06	0.335	0.564	−0.194	90	0.847
4	1040	43.86	1331	56.14	1:1.28	0.579	0.449	−0.596	70	0.553
5 (I)	63	43.75	81	56.25	1:1.29	0.487	0.488	−0.585	70	0.560
5 (II)	49	38.89	77	61.11	1:1.57	7.891	0.007 *	−1.211	52	0.231
6	76	43.93	97	56.07	1:1.28	0.444	0.508	−0.350	52	0.728

* *p* = 0.007 < 0.05, the homogeneity of variances cannot be assumed.

## Data Availability

The original contributions presented in the study are included in the article, further inquiries can be directed to the corresponding author.

## Supplementary Materials

The following supporting information can be downloaded at: https://www.mdpi.com/article/10.3390/insects15100738/s1, Figure S1: Stages of *D. frumenti*: (a) egg, (b) larva and detail of its powerful mandibula, (c) pupa and (d) adult with detail of sexual differentiation on the basis of the face (credits: (a) Santiago, M. and (b)–(d) Peña, A.).; Figure S2: Direct damage by *D. frumenti* to a palm tree: (a) exit holes, (b) presence of gummy exudates at the entrance of the galleries, (c) galleries in a cross section of the rachis of a leaf, (d) lateral desiccation at the base of the leaves and (e) collapse of the basal rings of the palm leaves (credits: (a) and (b) Peña, A. and (c)–(e) Ramos Cordero, C.).
