# Supplementary material for: Effect of Commercial Trap Design and Location on Captures of Diocalandra frumenti (Fabricius) (Coleoptera: Dryophthoridae) on Palm Trees
Source: Insects. 2024 Sep 25;15(10):738. doi: 10.3390/insects15100738 (PMC11508261; doi:10.3390/insects15100738)
Supplement: Supplementary file 1 [file insects-15-00738-s001.zip › insects-3209360-supplementary.pdf]

Supplementary information

Carina Ramos-Cordero [1,2*], Elena Seris-Barrallo [1,3], Sandra Vacas [4], Vicente Navarro-Llopis [4] and Estrella M. Hernández-Suárez [2]

1 Dirección General de Agricultura, Consejería de Agricultura, Ganadería y Pesca del Gobierno de Canarias, Santa Cruz de Tenerife, Avda. José Manuel Guimerá, 10, Edif. Servicios Múltiples II, Planta 4ª, 38071, Santa Cruz de Tenerife, España; seris.elena@inia.csic.es (E.S.-B.)

2 Unidad de Protección Vegetal, Instituto Canario de Investigaciones Agrarias (ICIA), Crta. El Boquerón, s/n, 38270, La Laguna, España; ehernand@icia.es (E.H.-S.)

3 Unidad de Productos Fitosanitarios, Instituto Nacional de Investigación y Tecnología Agraria y Alimentaria, Crta. de La Coruña, km 7,5, 28040, Madrid, España; seris.elena@inia.csic.es (E.S.-B.)

4 Centro de Ecología Química Agrícola, Instituto Agroforestal del Mediterráneo, Universidad Politécnica de València, Camino de Vera, s/n, 46022, Valencia, España; sanvagon@ceqa.upv.es (S.V.); vinallo@ceqa.upv.es (V.N.-L.)

* Correspondence: ramoscorderocarina@gmail.com

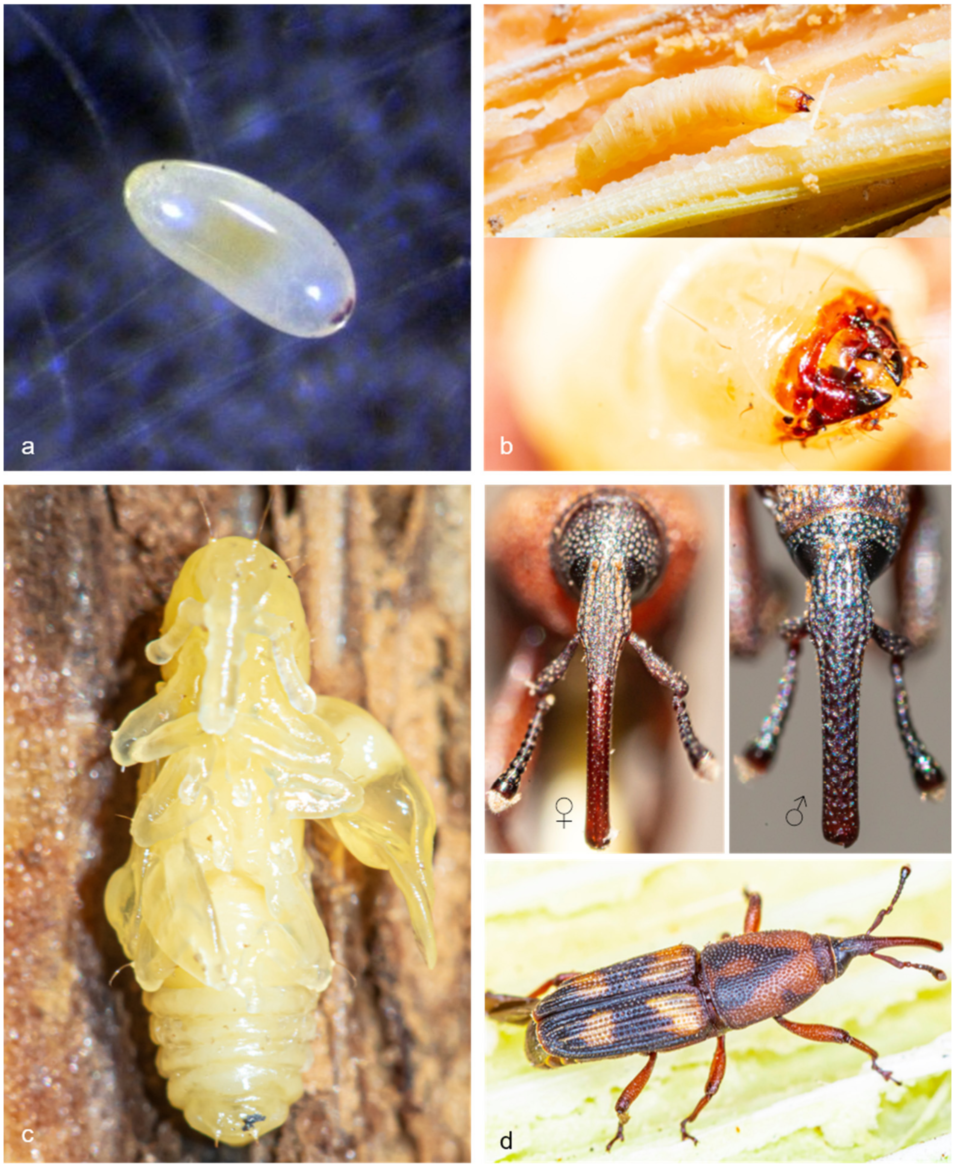

Figure S1. Stages of *D. frumenti*: a) egg, b) larva and detail of its powerful mandibula c) pupa and d) adult with detail of sexual differentiation on the basis of the face (Credits: a) Santiago, M. and b-d) Peña, A.).

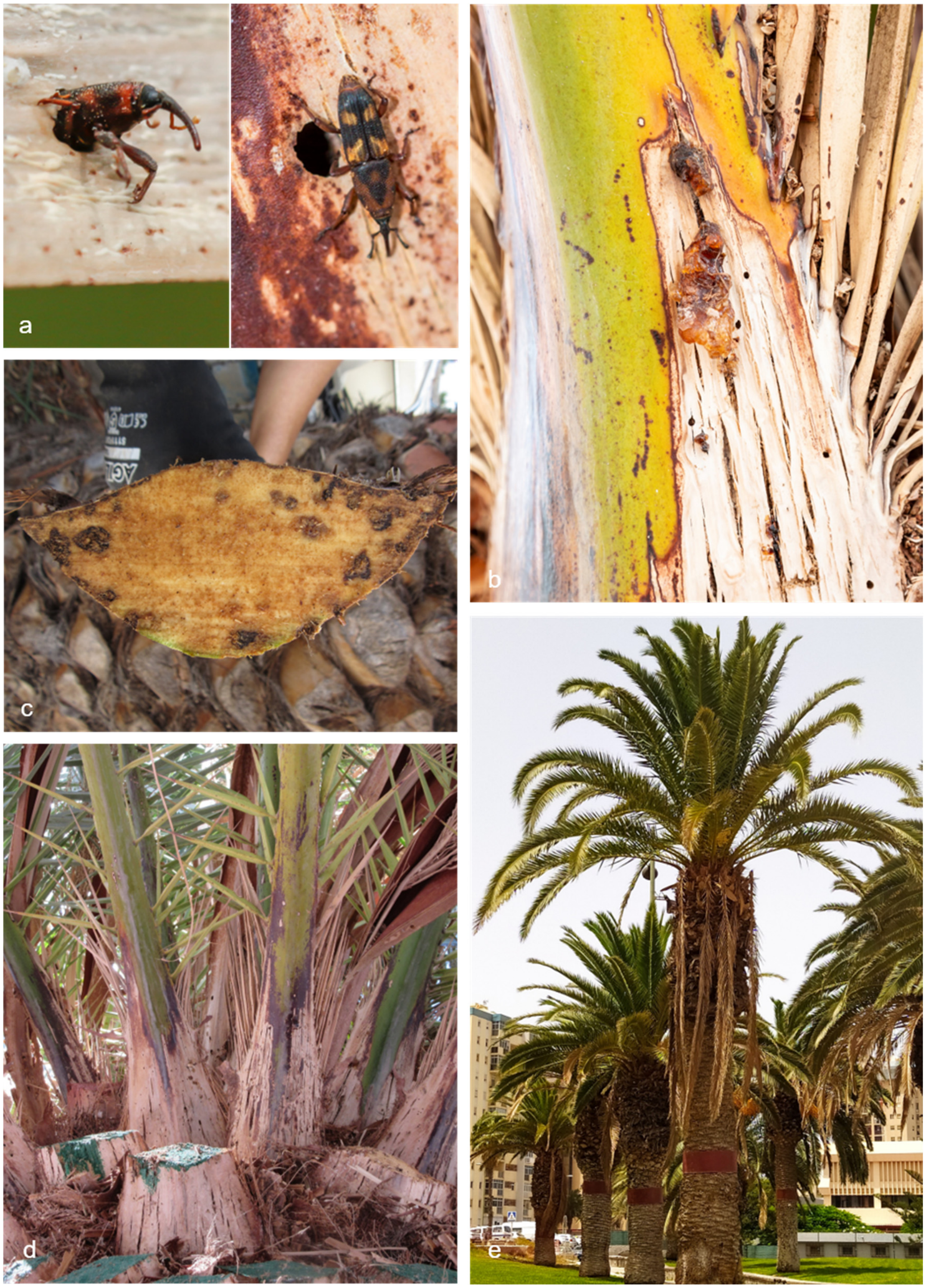

Figure S2. Direct damage by *D. frumenti* in a palm tree: a) exit holes, b) presence of gummy exudates at the entrance of the galleries, d) galleries in a cross section of the rachis of a leaf, e) lateral desiccation at the base of the leaves and g) collapse of the basal rings of the palm leaves (Credits: a y b) Peña, A. and c-e) Ramos Cordero, C.).
